# Do specialty registrars change their attitudes, intentions and behaviour towards reporting incidents following a patient safety course?

**DOI:** 10.1186/1472-6963-10-100

**Published:** 2010-04-23

**Authors:** José D Jansma, Dorien LM Zwart, Ian P Leistikow, Cor J Kalkman, Cordula Wagner, Arnold B Bijnen

**Affiliations:** 1 Foreest Medical School, Medical Center Alkmaar, Wilhelminalaan 12, 1815JD Alkmaar, the Netherlands; 2 EMGO Institute for Health and Care Research, VU University Medical Center, Van der Boechorststraat 7, 1081BT Amsterdam, the Netherlands; 3 University Medical Center Utrecht Patient Safety Center, Heidelberglaan 100, 3584CX Utrecht, the Netherlands; 4 University Medical Center Utrecht, Julius Center for Health Sciences and Primary Care, Heidelberglaan 100, 3584CX Utrecht, the Netherlands; 5 NIVEL Netherlands Institute for Health Services Research, Otterstraat 118 - 124, 3500BN Utrecht, the Netherlands; 6 VU University Medical Center, Institute for Education and Training, Van der Boechorststraat 7, 1081BT Amsterdam, the Netherlands

## Abstract

**Background:**

Reporting incidents can contribute to safer health care, as an awareness of the weaknesses of a system could be considered as a starting point for improvements. It is believed that patient safety education for specialty registrars could improve their attitudes, intentions and behaviour towards incident reporting. The objective of this study was to examine the effect of a two-day patient safety course on the attitudes, intentions and behaviour concerning the voluntary reporting of incidents by specialty registrars.

**Methods:**

A patient safety course was designed to increase specialty registrars' knowledge, attitudes and skills in order to recognize and cope with unintended events and unsafe situations at an early stage. Data were collected through an 11-item questionnaire before, immediately after and six months after the course was given.

**Results:**

The response rate at all three points in time assessed was 100% (n = 33). There were significant changes in incident reporting attitudes and intentions immediately after the course, as well as during follow-up. However, no significant changes were found in incident reporting behaviour.

**Conclusions:**

It is shown that patient safety education can have long-term positive effects on attitudes towards reporting incidents and the intentions of registrars. However, further efforts need to be undertaken to induce a real change in behaviour.

## Background

Patients are at risk of suffering harm as a consequence of adverse events during their treatment [1-5]. Research has shown that a large proportion of these adverse events was considered preventable and was related to human behaviour [2,3,5-7].

Voluntary and non-punitive reporting of unintended or unexpected events, which might or did lead to harm for one or more patients, can be a valuable method both to gain insight into the occurrence and causes of incidents and to identify risk factors which should be acted upon to improve safety [8-10]. Systems for reporting incidents in other high-risk sectors, such as the aviation and the petrochemical industry, have demonstrated the usefulness of this method as they resulted in measurably safer processes [8]. There are three principal conditions for creating an effective reporting system: 1) health care workers must be aware of the importance of reporting incidents; 2) they need to know how to report an incident; and 3) they must be able to recognize risky situations [11]. Patient safety education is perceived as a major incentive to achieving an active reporting culture and thereby contributing to a reduction of risks in patient care. When the extent of adverse events in health care became visible, the need for patient safety education was adopted in policies in many countries [12-15].

For several reasons it is expected that patient safety education for registrars can lead to particularly valuable results. Firstly, registrars provide much of the direct patient care [16]. Secondly, they are considered a fragile link in the care process. Research has revealed that a lack of work experience and high pressure of work among registrars increases risky situations [17,18]. Besides, research showed that medical trainees' knowledge of patient safety across a broad range of training levels, degrees and specialties was limited [19] and that doctors have a relatively low rate of incident reporting [20]. A final argument for training registrars in patient safety is that they are considered to be a group which can achieve long-lasting benefits, as these physicians are at the beginning of their career and they are the medical specialists of the future.

Although medical education has been paying greater attention to patient safety, only a few studies have been conducted to evaluate the effectiveness of patient safety courses. Most of these studies focussed on medical students and/or did not measure long-term effects [21-25]. Only one of these studies used incident reporting attitudes and behaviour as the outcome measure. However, in that study long-term educational effects were not evaluated [21].

The objective of the current study is to examine the long-term impact of a two-day patient safety course for registrars from different specialties on their attitudes, intentions and behaviour towards the voluntary reporting of incidents. According to the Theory of Planned Behaviour, which is strongly supported by empirical evidence, attitudes and intentions, together with subjective norms, are the components that can predict behaviour [26].

## Methods

### Course

A patient safety course was designed according to the process-oriented teaching model of Vermunt and Verloop (1999) [27]. The goal was to increase specialty registrars' knowledge, attitudes and skills in order to recognize and cope with unintended events and unsafe situations at an early stage. The curriculum was delivered by external speakers, as well as by employees of the Patient Safety Center and the general practitioners vocational training of the University Medical Center Utrecht, the Netherlands. The course, which was part of a series of multidisciplinary courses for the registrars, consisted of two consecutive days. A mix of educational methods [28] was utilized to create an optimal learning environment with an interactive character. An overview of the course content is presented in table 1.

**Table 1:** Content of the patient safety course

Themes	Objectives	Educational methods	References
1. Kick-off	Get to know each other and explore the existing prejudices concerning errors and incidents within the group. Make agreements on confidentiality.	Plenary session; group discussions; case presentations.	

2. Background of patient safety	Outline the history, how the concepts have been determined and the current national and international positions of patient safety.	Plenary session; group discussions.	Baker *et al*. 2004 [32] Willems 2004 [10]

3. Human Error	Give insight into human factors as a major source for learning from errors and incidents with a link to safety in the aviation industry.	Plenary session; group discussions.	Dekker 2002 [37] Casey 2008 [38]

4. Proceeding after an incident	Practice skills on how to approach colleagues after an incident has occurred.	Experiential learning in small groups; interview, role-play, reflection	Gallagher *et al*. 2003 [39] Chan *et al*. 2005 [40] Gawande 2002 [41] Newman 1996 [42]

5. Medico-legal aspects of critical incidents	Transfer knowledge about the medico-legal aspects of critical incidents in health care.	Plenary session; group discussions; summative knowledge test.	Legemaate *et al*. 2007 [43]

6. Learning from errors	Explain and apply methods for analysing incidents and processes such as analysing the root causes and initiating risk analysis.	Plenary session; group discussions; experiential learning in small groups.	Habraken *et al*. 2009 [44] van Vuren 1999 [45]

7. A view from the sharp end	Give an explanation of the potential risks that can be found in the design of systems and products.	Plenary session; group discussions.	Barach and Small 2000 [8]

8. Contact with a patient after an incident	Practice difficult conversations focused on how to approach a patient after an incident has occurred.	Guided role-play in small groups with experienced actors.	Duclos *et al*. 2005 [46] Gallagher *et al*. 2005 [47]

9. Tips and Tools for daily practice	Convert the knowledge and experiences gathered into initiatives for improving safety in daily practice.	Plenary session; group discussions.	Jagsi *et al*. 2005 [17] Volpp *et al*. 2003 [16]

### Data collection

Data were collected before the course, immediately after the course and six months later. The first two assessments took place at the site of the course, where data were collected by means of an electronic voting system. For these surveys, questions were displayed on a projection screen and participants were asked to answer these questions anonymously by using a voting device. As soon as a question was answered by everyone, the group result was portrayed in a graph on the screen. Approximately six months after the course, follow-up data collection was initiated by e-mail contact with the participants. The Scientific Committee of the VU University Medical Center, the Netherlands provided a waiver for this study. National rules and regulations for health services research were followed.

### Questionnaire

The same set of eleven questions was used for all three assessments (see table 2 and 3).

**Table 2:** Results of vignette questions (n = 33)

	Pre-course			Post-course			Follow-up			
		
Do you consider the following events worth a report?	No	Cannot Decide	Yes	No	Cannot Decide	Yes	No	Cannot Decide	Yes	Significance
**1. You bring the wrong patient to the operating room, you notice your mistake in time and pick up the right person.**	16 (48%)	11 (33%)	6 (18%)	12 (36%)	5 (15%)	16 (48%)	9 (27%)	12 (36%)	12 (36%)	p = 0.049

**2. At the start of your shift you notice that Mr. B's heparin pump is adjusted too high.**	12 (36%)	12 (36%)	9 (27%)	4 (12%)	6 (18%)	23 (70%)	4 (12%)	3 (9%)	26 (79%)	p < 0.001

**3. You requested, urgently, the results of a laboratory test but you received them much too late.**	19 (58%)	6 (18%)	8 (24%)	10 (30%)	5 15%)	18 (55%)	3 (9%)	13 (39%)	17 (52%)	p < 0.001

**4. The treatment policy of Mrs. X changed, but so far there is no notification of this in her status.**	28 (85%)	2 (6%)	3 (9%)	12 (36%)	10 (30%)	11 (33%)	9 (27%)	11 (33%)	13 (39%)	p < 0.001

**5. You notice that the ampoules are not placed as usual, you were not informed about a change in policy.**	30 (91%)	1 (3%)	2 (6%)	17 (52%)	7 (21%)	9 (27%)	8 (24%)	17 (52%)	8 (24%)	p < 0.001

**6. On hindsight it became clear that the diagnosis of Mr. M was wrong, the patient did not experience any disadvantages.**	23 (70%)	8 (24%)	2 (6%)	14 (42%)	9 (27%)	10 (30%)	18 (55%)	5 (15%)	10 (30%)	NS

**Table 3:** Incident reporting attitudes, intentions and behaviour (n = 33)

	Pre-course			Post-course				Follow-up			
	
	No	Cannot Decide	Yes	No	Cannot Decide	Yes	Significance Pre-course vs Post-course	No	Cannot Decide	Yes	Significance Pre-course vs Follow-up
**7. Do you think it is important for registrars to report incidents where there is no harm done to the patient(s)?**	7 (21%)	0	26 (79%)	5 (15%)	0	28 (85%)	NS	0	2 (6%)	31 (94%)	p = 0.005

**8. Do you think it is important for registrars to report incidents where there is harm done to the patient(s)?**	2 (6%)	0	31 (94%)	0	0	33 (100%)	NS	0	0	33 (100%)	NS

**9. Are you seriously considering reporting incidents within the next six months?**	10 (30%)	0	23 (70%)	3 (9%)	0	30 (91%)	p = 0.030	0	2 (6%)	31 (94%)	p = 0.011

**10. Are you planning to start reporting within the next month?**	12 (36%)	0	21 (64%)	6 (18%)	0	27 (82%)	NS	5 (15%)	4 (12%)	24 (73%)	p = 0.045

**11. Have you reported a incident within the last six months?**	20 (61%)	0	13 (39%)					19 (58%)	0	14 (42%)	NS

The first six so-called *vignette questions *were developed by two of the authors (CW&ABB) and were based on their experiences in health care and patient safety research. These vignettes were intended to gain insight in the registrars' attitudes towards incident reporting in specific situations. Incident reporting in this setting meant voluntary reporting of incidents by filling out a digital registration form and sending it to a specific hospital committee. Subsequently, these reports are analysed by the committee and, if necessary, the committee gives advice for improvements that should prevent recurrence of the incident. The patient safety course stressed the importance of making a report of all unintended or unexpected events which might or did lead to harm for one or more patients. Therefore it would have been correct to consider all the cases in the questionnaire worthy of a report.

Questions 7-11 focused on attitudes, intentions and behaviour towards reporting incidents and were based on the questionnaire of Coyle *et al. *(2005), who measured the impact of a patient safety educational programme exclusively for family practice residents [21].

In the present study three adjustments were made to Coyle's questionnaire. Firstly, three response options were given (Yes/Cannot decide/No) instead of the two options given by Coyle (Yes/No). Secondly, the measurement of attitudes regarding the importance of incident reporting was divided into two different outcomes (*with *and *without *harm for the patient). Lastly, while Coyle asked his respondents when they had started reporting incidents, this question was omitted for this study. This was because, unlike Coyle's respondents, a major part of the current registrars had already started reporting incidents before they attended the course.

The health psychology Stages of Change Model [29], which explains intentional behavioural changes, was used as a theoretical framework for a major part of Coyle's questionnaire. This model distinguishes four consecutive stages related to behavioural change: pre-contemplation, contemplation, preparation and action [29]. Coyle gave definitions for these categories that were also incorporated into this study. Participants in the pre-contemplation stage are not currently reporting incidents and are not considering doing so in the next six months. 'Contemplators' on the other hand, are also not currently reporting incidents, but are planning to do so within the next six months. Individuals in the preparation stage are not currently reporting incidents but in these cases are planning to start reporting within the next 30 days. In the action stage participants have begun reporting incidents within the last six months [21].

### Analysis

Data were analysed using SPSS version 15.0. For each question the percentages of the three different categories of answer (Yes/Cannot decide/No) were calculated. The significance of the differences between the three points in time was determined with Pearson Chi-Square tests (p < 0.05). To enable the use of the Fisher's exact test, for analysing questions 7-12, two answer categories were created: 1) Yes/Cannot decide and 2) No. To analyse the six vignette questions, a sum variable was created.

After the patient safety course we measured significant positive changes in attitudes, these changes remained stable over time, so no significant changes were found between the post-course and follow-up measurement. Therefore there was no linear trend in time. Regression analysis showed there was no correlation between the measurement and the questionnaires' outcomes (R Sq was only 0.1).

## Results

### Participants

In December 2006, 33 registrars from the district of Utrecht attended the patient safety course. Table 4 shows the characteristics of the study participants. For all three assessments the response rate was 100% (n = 33). The median response time at follow-up was 16 days after follow-up data collection started.

**Table 4:** Characteristics of the study participants

Age, years	
Range	30 - 35
Median age	32
	
Sex, n (%)	
Male	12 (36)
Female	21 (64)
	
Year of residency during course, n (%)	
First	4 (12)
Between first and last	18 (55)
Last	10 (30)
Missing	1 (3)
	
Discipline, n (%)	
General practice	10 (30)
Anaesthesiology	10 (30)
Dermatology	4 (12)
Internal medicine	4 (12)
	
Other	5 (15)

### Baseline

**Figure 1:**
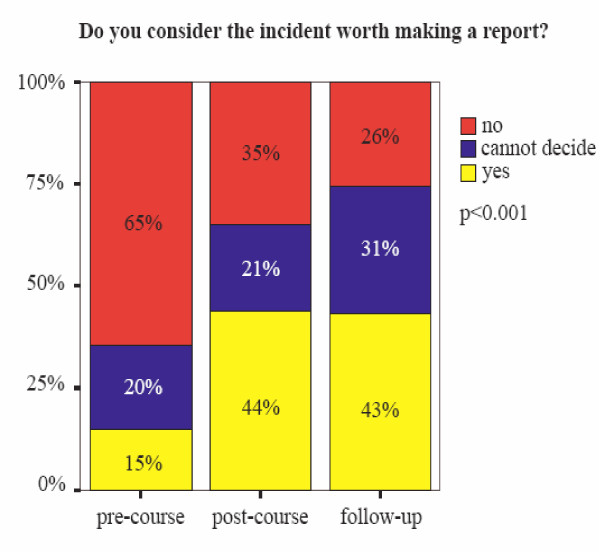
**Frequency of the answers to the six vignette questions in sum**.

At the first measurement a minority of the registrars considered the vignettes worth reporting (figure 1). A majority of respondents judged reporting by registrars important for incidents with harm, as well as incidents without harm for the patient(s). A majority of the participants recorded an intention to report incidents, though less than half of the registrars indicated that they have reported an incident within the last six months. An overview of the exact answers given at the separate measurements is given in table 2 and 3.

### Changes in reporting attitudes (question 1 - 8)

After the course all vignettes more often were considered worth reporting, in five out of six the changes were significant (table 2). Analysis of the constructed sum variable of the six vignette questions shows that immediately after the course, as well as during follow-up, there were significant changes in attitudes towards reporting incidents (p < 0.001). After attending the patient safety course, registrars more often considered it worth reporting the incidents proposed in the questionnaire (figure 1).

Measurements of the registrars' views concerning the necessity of reporting incidents *without *harm (question 7) or *with *harm (question 8) to the patient both demonstrate that after the course more registrars judged reporting by registrars to be important. These changes were not only significant for question 7 when the pre-course measurement was compared to the follow-up measurement.

### Changes in reporting intentions (question 9&10)

Intentions to report incidents within the next six months (table 3, question 9) showed an increase immediately after the course as well as during follow-up. Plans to report incidents within the next month also increased, but this change only reached statistically significant levels when pre-course and follow-up measurements were compared (table 3, question 10).

### Changes in reporting behaviour (question 11)

At the follow-up the number of registrars who declared they had reported an incident in the last six months had increased by one, this change was not significant.

## Discussion

Incident reporting by health care workers is considered an important step towards improving patient safety. This study focussed on a course that aimed at increasing the knowledge, attitudes and skills of registrars, in order to improve the safety of patients. The objective of this study was to assess the impact of the course on attitudes, intentions and behaviour of the participants towards reporting incidents.

The study led to three major results. Firstly, after attending the course the registrars' ability to assess what kind of incident deserves reporting had improved. Secondly, after the course, intentions to report incidents increased significantly (*contemplation *and *preparation stage*). Thirdly, no significant changes were found in incident reporting behaviour (*action stage*).

The first two results indicate that the course had a positive impact on registrar attitudes. The elements of the course that may particularly have contributed to this positive impact were the group discussions about incident reporting and experiences with incidents that were outlined by teachers and registrars. However, as the third result shows, a discrepancy remains between registrars' intentions to report incidents and their behaviour. This discrepancy was also found in other studies [21,30].

### Reporting barriers

It is unlikely that the participants did not report incidents because of the absence of these in their work, as research revealed that registrars are regularly involved in incidents [1-5,31-33]. This study did not make an inventory of the possible barriers that might discourage incident reporting among the registrars. However, other publications [20,21,34] suggest that there are several barriers, related to human as well as system factors, that could hinder incident reporting. These included: time constraints, complex reporting systems, no perceived benefits, forgetfulness, no encouragement from the faculty, no timely and high quality feedback on medical event reports, risks to one's career and personal reputation and a lack of knowledge of what to report.

Apart from the individual barriers, the participants in this study constitute just a small proportion of the entire population of the health care workers in their district. The impact of education on behavioural change may suffer if only a small part of the team members are trained instead of the entire health care team [35]. To achieve permanent changes in engrained behavioural patterns it is important to focus not only on individual attitudes and intentions, but also on a stimulating environment, including hospital culture and patient safety policies [26].

It is expected that explanations for the absence of reports may vary between different settings, as every organisation has its own particular policy and culture. For example, at the time of this study, incident reporting in Dutch general practices was rare, and incident reporting systems were mostly not available [36]. Research should be conducted in order to reveal the barriers that these registrars experienced and that discouraged them from reporting incidents.

### Study limitations

This study focussed on one group of just 33 registrars. This number of participants might have been too small to detect changes in behaviour. Furthermore, the results could be very much dependent of the specific setting in this study hospital. Another limiting factor is that the outcomes were based on the perception of the respondents, which might provoke social desirability bias. The fact that the first two measurements were carried out anonymously and that the data were collected by an independent researcher hopefully has minimized this bias. Also, a 'testing effect' may have influenced the internal validity of this study. If respondents are repeatedly asked to fill out a questionnaire, their score might improve each subsequent time because of practice, familiarity or awareness. However, we believe that the effect of possible testing bias was minimal in this study because the second and third measurements were about six months apart. Lastly, the questionnaire that was used was not validated. Although a major part of the questionnaire had been used before elsewhere [21], its validity towards attitudes and intentions still remains to be proven.

### Directions for future research

Thus, a change in attitudes and intentions is not sufficient to induce a real change in behaviour. Therefore, barriers, perceived by registrars to discourage the reporting of incidents, should be analyzed. This can be considered a starting point in overcoming these barriers. A further step should be taken to ensure the benefits of patient safety education to continue throughout the educational career. Investigating the role of the participants' characteristics is recommended for future evaluations of patient safety education. Previous research showed that the degree of knowledge about patient safety varied significantly depending on characteristics such as the year of training, specialty, gender and age [19].

## Conclusions

This study showed that multispecialty patient safety education can have positive effects, both immediately and in the long-term, on attitudes towards reporting incidents and the intentions of specialty registrars. Therefore, patient safety education should be integrated within medical education. Although registrars in this study judged that reporting incidents by registrars is important, there is a gap between the registrars' intentions to report incidents and their actual behaviour. Therefore, further steps are needed to stimulate a real change in behaviour.

## Competing interests

The authors declare that they have no competing interests.

## Authors' contributions

JDJ collected and analyzed the data, and wrote the manuscript. DLMZ, IPL and CJK drew up the educational approach, and co-wrote the manuscript. CW designed the study, collected the data, and co-wrote the manuscript. ABB designed the study, collected the data and co-wrote the manuscript. All authors read and approved the final manuscript.

## Authors' information

José D. Jansma, MSc, is a PhD student of the research programme Patient Safety in the Netherlands. She is working for the Foreest Medical School of the Medical Center Alkmaar, the Netherlands, and the Department of Public and Occupational Health, EMGO Institute, VU University Medical Center, the Netherlands.

Dorien L.M. Zwart, MD, is general practitioner and assistant professor of the general practitioner vocational training of the Julius Center for Health Sciences and Primary Care, University Medical Center Utrecht. In addition, she is researcher at the University Medical Center Utrecht Patient Safety Center, the Netherlands.

Ian P. Leistikow, MD, is coordinator of the University Medical Center Utrecht Patient Safety Center, the Netherlands.

Cor J. Kalkman, MD, PhD, is professor in anaesthesiology and head of the University Medical Center Utrecht Patient Safety Center, the Netherlands.

Cordula Wagner, MA, PhD, is head of research area 'Quality and Organisation of hospital- and long-term care' at NIVEL Netherlands Institute for Health Services Research. In addition, she is supervisor of the Dutch research programme on patient safety in hospitals at the department of public and occupational health/EMGO Institute, VU Medical Center, the Netherlands.

Arnold B. Bijnen, MD, PhD, is a general gastro-intestinal surgeon at the Medical Center Alkmaar. He has been appointed professor of surgery at the VU University Medical Center for teaching and postgraduate training.

## Pre-publication history

The pre-publication history for this paper can be accessed here:

http://www.biomedcentral.com/1472-6963/10/100/prepub
